# Let’s Talk About Each Other: Neural Responses to Dissenting Personality Evaluations Based on Real Dyadic Interactions

**DOI:** 10.1177/0956797621995197

**Published:** 2021-06-08

**Authors:** Sebastian Schindler, Anne Höhner, Robert Moeck, Maximilian Bruchmann, Thomas Straube

**Affiliations:** 1 Institute of Medical Psychology and Systems Neuroscience, University of Münster; 2 Otto Creutzfeldt Center for Cognitive and Behavioral Neuroscience, University of Münster

**Keywords:** EEG, ERP, social interaction, real social evaluation, dyadic interaction, self-incongruence, preregistered

## Abstract

Dyadic interactions are associated with the exchange of personality-related messages, which can be congruent or incongruent with one’s self-view. In the current preregistered study (*N* = 52), we investigated event-related potentials (ERPs) toward real social evaluations in order to uncover the neural mechanisms underlying the processing of congruent and incongruent evaluative feedback. Participants interacted first, and then during an electroencephalogram (EEG) session, they received evaluations from their interaction partner that were either congruent or incongruent with their own ratings. Findings show potentiated processing of self-related incongruent negative evaluations at early time points (N1) followed by increased processing of both incongruent negative and positive evaluations at midlatency time windows (early posterior negativity) and a prioritized processing of self-related incongruent positive evaluations at late time points (feedback-related P3, late positive potential). These findings reveal that, after real social interactions, evaluative feedback about oneself that violates one’s self-view modulates all processing stages with an early negativity and a late positivity bias.

During communication, we constantly exchange and share evaluations, including personality-related judgments (for a recent review, see Falk & Scholz, 2018). The motivational importance of such evaluations is associated with pronounced modulations of early and late neural processes of feedback processing, which have been found in event-related potential (ERP) studies (e.g., Rohr & Abdel Rahman, 2018; Schindler et al., 2015). Several studies have revealed that the self-relatedness of the evaluation and the relevance of the interaction partner affect early and late components of the ERP, which are associated with sensory processing (P1, N1; e.g., Bayer et al., 2017), early attentional selection (early posterior negativity [EPN]; e.g., Schindler et al., 2015), and sustained emotional processing and stimulus evaluation (late positive potential [LPP]; e.g., Herbert et al., 2011). Whereas negative feedback—especially unexpected negative feedback—has been shown to affect early responses (P1 and N1; see Harrewijn et al., 2018; Schindler, Vormbrock, & Kissler, 2019), the effect of positive feedback is expected to affect later processing stages. It has recently been suggested that healthy participants exhibit positively biased self-updating processes, leading to a more elaborative processing of self-serving information (e.g., Korn et al., 2012; Sharot & Garrett, 2016). Such elaborative stimulus processing, including self-relational processing, integration, and emotion-regulation processes, more heavily influence late processing stages (see Hajcak et al., 2010; Schupp et al., 2006).

However, how recipients process evaluations during social interactions also depends on their expectations and on their self-concepts (see Kissler, 2020). The detection and integration of discrepant evaluations given by the interaction partner are a key aspect here (Shrauger & Schoeneman, 1979). Thus, the recipients’ own views and the way they process the received feedback matter strongly in social settings (Behrens et al., 2008), leading to the question of how neural correlates of feedback processing are modulated by the congruence and incongruence between the self-view and the evaluation given by the interaction partner. Here, performance and social-feedback studies identified two ERP components of special interest—the feedback-related negativity (FRN) and the feedback-related P3 (Becker et al., 2014; Kujawa et al., 2014; see also Pfabigan et al., 2011; van der Molen et al., 2017; Veen et al., 2016). The FRN is measured as a relative negativity after feedback, where negative feedback elicits a more negative deflection and positive feedback elicits more positive amplitudes (Hajcak et al., 2006). These relatively more positive-going amplitudes are also described as *reward positivity* (Proudfit, 2015). Studies have shown more negative FRN responses for social rejections but more positive feedback-related P3 responses for social acceptance (Kujawa et al., 2014; van der Molen et al., 2017; Veen et al., 2016). Surprisingly, there is a lack of research on neural activations during the processing of congruent or incongruent personality-related messages—that is, the judgment of the interaction partner compared with one’s own personal evaluation. Furthermore, studies are needed to investigate how real social interactions affect neuronal responses, in accordance with a recent call for a second-person neuroscience from researchers positing that real interactions are necessary to understand social cognition (Caruana et al., 2017; Schilbach et al., 2013) and advocating real-life interactive paradigms (Shamay-Tsoory & Mendelsohn, 2019).

In this preregistered study (https://osf.io/9eqvy), we investigated ERPs during the processing of congruent and incongruent personality-related messages based on real dyadic interactions. We predicted that self-related evaluations should increase ERPs across all processing stages, whereas incongruent evaluations were expected to strongly affect FRN and feedback-P3 amplitudes. Importantly, we predicted an early amplification of self-related incongruent negative evaluations (P1, N1, EPN), which we expected would be followed by stronger effects of self-related positively dissenting evaluations during late time windows (feedback P3, LPP).

**Statement of Relevance**

Exchanging social evaluations about each other is a central aspect of human communication. These opinions of others can differ from our own. In this study, participants were shown adjectives describing personality traits, and they rated the degree to which the adjectives applied to themselves and their interaction partners. We showed participants both evaluations and measured how their brains reacted to violations of their own ratings. EEG recordings allowed us to assign these violations to different stages of information processing. Participants’ own evaluations were more intensely processed across the entire processing stream than their evaluations about others. Interestingly, neuronal responses to self-incongruent evaluations showed an early negative bias, followed by a late positive bias. This late response to more positive evaluations might explain previously observed positively biased self-updating after social feedback. Thus, when you are faced with incongruent evaluations given by others, it matters when it is about yourself. This incongruence is detected early and shows specific temporal-valence biases.

## Method

### Participants

Fifty-two participants (26 dyads; 13 male-male, 13 female-female) were recruited at the University of Münster. All gave informed consent and received €10 per hour for participation. Six participants were excluded because of extensive artifacts in the recorded electroencephalogram (EEG). We investigated dyads only of the same sex to control for possible confounds of same- versus other-sex dyads. The final sample (*N* = 46; 23 male, 23 female) consisted of native German speakers; all were right-handed, had normal or corrected-to-normal vision, and reported no previous or current neurological or psychiatric disorders. On average, participants were 23.70 years old (*SD* = 3.45).

### Stimuli

A stimulus set of 120 adjectives (60 negative, 60 positive) was selected. These adjectives had previously been rated by 22 students in terms of valence and arousal using the Self-Assessment Manikin (SAM; Bradley & Lang, 1994). We also created an analogous SAM scale for concreteness ratings, referring to the appropriateness of an adjective to characterize a person. These raters had been instructed to consider the adjectives’ valence, arousal, and concreteness in an interpersonal evaluative context. The selected adjectives were strictly matched with regard to their ratings and linguistic properties (word length, frequency, familiarity, and regularity), and eventually differed only in rated valence (see Table 1).

**Table 1.** Comparisons of Negative and Positive Adjectives Using Independent-Samples *t* Tests

Variable	Negative adjectives (*n* = 60)	Positive adjectives (*n* = 60)	*t*(118)	*p*
Valence	3.01 (0.58)	7.25 (0.62)	38.68	< .001
Arousal	4.65 (0.71)	4.57 (0.71)	0.65	.515
Concreteness	3.13 (0.67)	2.98 (1.03)	0.97	.334
Word length	8.95 (2.68)	9.12 (2.98)	0.34	.731
Word frequency (per million)	465.23 (728.19)	471.88 (783.92)	0.05	.962
Familiarity (absolute)	16,229.55 (32,065.90)	19,923.33 (42,684.62)	0.54	.593
Regularity (absolute)	212.90 (398.68)	292.72 (447.57)	1.03	.304
Initial trigram frequency (absolute)	264,114.77 (508,249.74)	237,740.53 (429,313.24)	0.31	.759
Coltheart’s neighborhood density (cumulative frequency)	2,780.72 (18,325.83)	2,678.58 (12,709.54)	0.04	.972

Note: Standard deviations are given in parentheses.

### Procedure

**Fig. 1.**
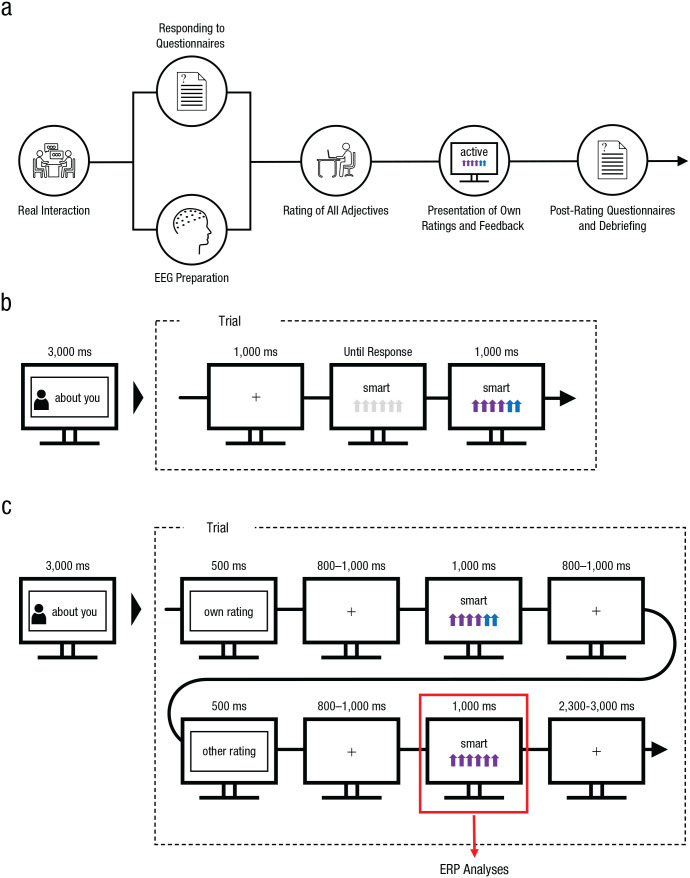
Experimental design (a) and trial sequence (b and c). The complete timeline of the experiment is shown in (a). Electroencephalograms (EEGs) were prepared while participants responded to pretest questionnaires. Next (b), participants rated how well each of a set of adjectives described themselves or their interaction partner. Ratings were made by selecting from 1 to 6 arrows, which changed color to indicate the participants’ selection (a rating of 4 is shown here). In the main experiment (c), participants’ own ratings were presented first, followed by the rating decisions made by the interaction partner. The stimulus used for event-related potential (ERP) analyses is highlighted.

Participants were recruited via mass mailing and informed that an actual short interaction with another participant would take place during the experimental session. Within dyads, only participants who did not know each other were allowed to participate. In the lab, the two participants interacted with each other on the basis of a short, structured interview consisting of five questions alternating between participants (for an overview, see Fig. 1a). These questions asked the participants to describe themselves and gave them 1 min to answer each question. After the interview, participants were prepared for the EEG recording in two separate laboratories. During this preparation phase, they responded to different questionnaires—demographics questions, the Beck Depression Inventory (Hautzinger et al., 2009) and the State-Trait Anxiety Inventory (Spielberger et al., 1999)—and evaluated their initial interaction. Then they were asked to evaluate themselves and the other participant on all 60 negative and 60 positive adjectives (see Fig. 1b). The recipient of the ratings (self-evaluation or evaluation of the interaction partner) switched every 10 trials, as indicated by a short instruction presented for 3,000 ms. Each new rating trial started with a fixation cross presented for 1,000 ms, after which a personality word and a 6-point rating scale were displayed. Participants then had to assess the fit of that adjective—with respect to themselves or their interaction partner—using a 6-point Likert-type scale consisting of a series of arrows. The display highlighted their rating by changing the number of arrows selected to purple and the arrows not selected to blue (e.g., for a rating of 4, the first four arrows would turn purple, and the remaining two arrows would turn blue). The color constellation was counterbalanced across trials and depicted the evaluation value, from 1 (*weakly applicable*) to 6 (*strongly applicable*).

The subsequent EEG session started when all ratings had been collected and the two evaluation files were uploaded into the presentation program (see Fig. 1c). Participants were informed that during the session, their previously collected rating would be presented first each time (*own evaluation*), followed by the evaluation collected from the other participant (*other evaluation*). The recipient of these ratings (reference: self-related, other-related) switched every 10 trials, and this switch was highlighted by a short instruction presented for 3,000 ms. Each trial started with a short written prompt (500 ms) indicating that the participant’s own rating would be presented first, followed a fixation cross for 800 to 1,000 ms and then by the given rating for 1,000 ms. After another 800- to 1,000-ms fixation cross, a second prompt (500 ms) reminded the participant that now their interaction partner’s evaluation would be presented. This information was again followed by a fixation cross (800–1,000 ms) and finally by the other person’s rating (for 1,000 ms). Each trial finished with the display of another fixation cross for 2,300 to 3,000 ms, after which the next trial started or a block change occurred. Stimulus presentation was conducted using Presentation software (Version 21.1; Neurobehavioral Systems, 2019). In total, the experiment took about 2.5 hr.

### EEG recording and analysis

EEG data were recorded from 64 active electrodes (BioSemi, Amsterdam, The Netherlands; for the electrode layout, see https://www.biosemi.com/headcap.htm; for exact coordinates, see https://www.biosemi.com/download/Cap_coords_all.xls). The recorded sampling rate was 512 Hz. During recording, BioSemi uses two separate electrodes as ground electrodes (a Common Mode Sense active electrode and a Driven Right Leg passive electrode). Four additional electrodes were used to measure horizontal and vertical eye movements. These additional electrodes were placed at the outer canthi of the eyes and below and above the left eye.

Preprocessing and statistical analyses were conducted using BESA Research (Version 6.0; BESA, 2014) and Electromagnetic Encaphalography Software (EMEGS; Version 2.8; Peyk et al., 2011). Data were filtered off-line with a 0.01-Hz high-pass forward filter and a 40-Hz low-pass zero-phase filter. Filtered data were segmented from 100 ms before the onset of the other-evaluation display to 1,000 ms after stimulus presentation (see Fig. 1c). The 100 ms before evaluation onset were used for baseline correction. Eye movements were corrected using the automatic eye-artifact correction method implemented in BESA (Ille et al., 2002). Remaining artifacts were rejected on the basis of absolute threshold (120 µV), gradient (75), and low signal change (0.01). Bad EEG sensors were interpolated using a spline-interpolation procedure. On average, 3.02 electrodes (*SD* = 1.77) were interpolated. For ERP trials that were retained, no main effect of reference was found, *F*(1, 45) = 0.02, *p* = .874, η_*p*_² = .001. For congruence, there were fewer trials with the same rating from both interaction partners, leading to a main effect of congruence, *F*(1.43, 64.53) = 7.27, *p* = .004, η_*p*_² = .139. Importantly, there was no interaction effect of reference and congruence, *F*(1.30, 58.53) = 0.53, *p* = .515, η_*p*_² = .012. With respect to congruency, more incongruent negative evaluations (33.66 trials) and incongruent positive evaluations (34.19 trials) than congruent evaluations (26.03 trials) were kept (both *ps* < .001). Incongruent evaluations did not differ significantly (*p* = .865). For total available trials, see the first section in Results.

### Statistical analyses

EEG scalp data were statistically analyzed using EMEGS. For the evaluations, we used 2 (reference: self-related, other-related) × 3 (congruency: other evaluation more negative than self-rating, congruent self-rating and other evaluation, other evaluation more positive than self-rating) repeated measures analyses of variance (ANOVAs) investigating the main effects of reference and congruence and their interaction. For the N1 and EPN, the factor channel-group laterality (left, right) was also included.

Effect sizes are given as η_*p*_^2^s (Cohen, 1988). Degrees of freedom were Greenhouse-Geisser corrected when Mauchly’s test indicated a violation of sphericity. To validate expected time windows and electrode clusters, we inspected ERPs collapsed across all conditions (Luck & Gaspelin, 2017). Further, for the EPN and LPP, we used ERPs collapsed across reference conditions, inspecting differences between the incongruent and congruent conditions. Time windows were segmented from 80 to 100 ms for P1 effects, from 130 to 180 ms for N1, from 200 to 280 ms for FRN, from 260 to 360 ms for EPN, from 300 to 400 ms for frontal feedback P3, and from 500 to 750 ms for LPP effects. The P1 was scored over an occipital cluster (O1, Oz, O2), and the N1 and EPN were derived from two symmetrical occipital clusters (P9, PO7, P7, P10, P8, PO8). The FRN and frontal P3 were extracted from a frontal cluster (F1, Fz, F2, FC1, FCz, FC2), and the LPP component was scored centrally (FC3, FC1, FCz, FC2, FC4, C3, C1, Cz, C2, C4, CP1, CPz, CP2, P1, Pz, P2). The hypotheses, experimental design, and analysis plan were preregistered (https://osf.io/9eqvy). For a second hypothesis on ERP modulations for all evaluations, including participants’ own evaluations, see Section B in the Supplemental Material available online. All raw data have been made publicly available via OSF (https://osf.io/cfts6/).

## Results

### Interaction ratings and evaluation congruency

For the evaluations, available trial numbers of self- and other-referent evaluations were obviously identical, *F*(1, 45) = 0.00, *p* = 1.0, η_*p*_² < .001, but there were more incongruent positive and negative evaluations than congruent evaluations, *F*(1.29, 58.16) = 7.35, *p* = .005, η_*p*_² = .140, post hoc *ps* < .001 (see Table 2). The number of incongruent negative and positive trials did not differ (*p* = .974). Further, there was no interaction of reference and congruency, *F*(1.26, 56.65) = 0.12, *p* = .794, η_*p*_² = .003.

**Table 2.** Mean Interaction Ratings

Rating	Self-related			Other-related		
	Incongruent negative	Congruent	Incongruent positive	Incongruent negative	Congruent	Incongruent positive
Absolute evaluation	43.87 (17.14)	33.34 (7.46)	42.70 (17.57)	43.00 (17.84)	33.00 (7.57)	43.91 (17.54)

Note: Standard deviations are given in parentheses.

Participants also provided ratings about the interaction and evaluation both before and after Time 2 receiving evaluations from the other participant (Times 1 and 2, respectively; see Table 3). Compared with a hypothetical scale mean value, these ratings indicated above-average sympathy for the other participant, honesty of their submitted evaluations, and pleasantness. After participants received evaluations, their ratings indicated above-average ratings for perceived correctness, motivation to attend to self-related evaluations, and pleasantness (see Table 3).

**Table 3.** Comparison of Partner Ratings Before and After Evaluations Were Received

Time and variable	*M*	One-sample *t* test	*p*
Before receiving evaluations (Time 1)			
Sympathy	4.42^a^ (0.58)	16.35^a^	< .001
Honesty	4.60^a^ (0.58)	18.51^a^	< .001
Valence	3.25^a^ (0.82)	2.29^a^	.027
After receiving evaluations (Time 2)			
Correctness	4.28^b^ (0.59)	14.70^b^	< .001
Motivation	4.56^b^ (0.63)	17.04^b^	< .001
Valence	3.74^b^ (0.79)	6.18^b^	< .001

Note: Standard deviations are given in parentheses. ^a^One rating data set was not collected, resulting in a sample size of 45. ^b^Three rating data sets were not collected, resulting in a sample size of 43.

### ERP results

#### P1 (80–100 ms)

For the P1, there were no significant main effects of reference or congruency and no significant interaction between reference and congruency (see Tables 4 and 5).

**Table 4.** Mean Amplitude (µV) for all Event-Related Potential Components

Component	Self-related			Other-related		
	More negative	Congruent	More positive	More negative	Congruent	More positive
P1	1.68 (4.09)	1.51 (4.13)	1.42 (3.71)	1.43 (3.94)	1.65 (3.79)	1.39 (3.72)
N1	−1.35 (1.99)	−0.93 (1.95)	−1.23 (1.99)	−0.90 (1.91)	−1.05 (2.01)	−1.16 (2.03)
EPN	−2.40 (2.37)	−1.79 (2.58)	−2.46 (2.62)	−1.79 (2.38)	−1.85 (2.46)	−2.03 (2.48)
FRN	0.28 (1.96)	0.08 (2.19)	0.56 (2.09)	0.10 (2.16)	0.07 (2.02)	0.07 (2.02)
Feedback P3	1.28 (2.39)	1.22 (2.42)	1.65 (2.39)	0.96 (2.11)	1.14 (2.12)	0.87 (2.21)
LPP	2.15 (1.51)	1.79 (1.32)	2.40 (1.53)	1.76 (1.29)	1.76 (1.30)	1.85 (1.46)

Note: Standard deviations are given in parentheses. EPN = early posterior negativity; FRN = feedback-related negativity; LPP = late positive potential.

**Table 5.** Results From Analyses of Variance for all Event-Related Potential Components

Component	Main effect of reference			Main effect of congruency			Interaction effect		
	*F*(1, 39)	*p*	η_*p*_²	*F*(1, 39)	*p*	η_*p*_²	*F*(2, 90)	*p*	η_*p*_²
P1	0.17	.685	.004	1.21	.302	.026	1.11	.336	.024
N1	2.13	.151	.045	1.91	.155	.041	**4.49**	**.014**	**.091**
EPN	**12.29**	**.001**	**.214**	**4.78**	**.011**	**.096**	**4.99**	**.009**	**.100**
FRN	**5.55**	**.023**	**.110**	.193	.151	.041	2.14	.124	.045
Feedback P3	**8.45**	**.006**	**.158**	0.41	.663	.009	**4.53**	**.013**	**.091**
LPP	**12.75**	**.001**	**.221**	**4.93**	**.009**	**.099**	**3.48**	**.035**	**.072**

Note: Significant effects are in boldface. EPN = early posterior negativity; FRN = feedback-related negativity; LPP = late positive potential.

#### N1 (130–180 ms)

**Fig. 2.**
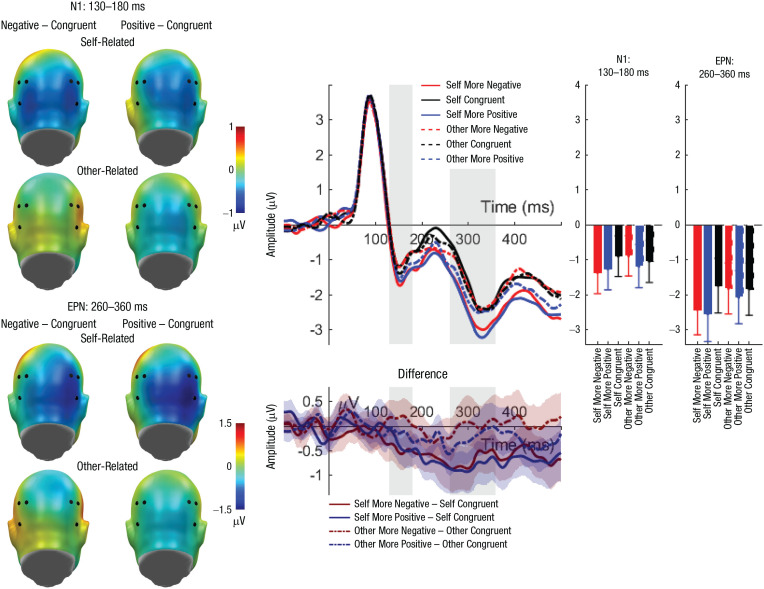
N1 and early posterior negativity (EPN): interaction effects of reference and evaluation congruency. Scalp topographies (left) depict differences between the negative and congruent conditions and positive and congruent conditions, separately for self-related and other-related evaluations. Black dots indicate positions of electrodes. The waveform graph (top middle) shows the time course of event-related potentials (ERPs) over the depicted average N1/EPN cluster, separately for incongruent positive, incongruent negative, and congruent self- and other evaluations. The gray shaded areas highlight the range of the N1 and EPN, respectively. The bar charts (right) show mean amplitudes for each evaluation type, separately for the N1 and EPN. Error bars represent 95% confidence intervals. The difference waveforms (bottom middle) show 95% bootstrapped confidence intervals of intra-individual differences in comparisons of interest.

Regarding the N1, no significant effects of reference or congruency were found. There was a significant main effect of laterality, *F*(1, 45) = 14.32, *p* < .001, η_*p*_² = .241; participants exhibited larger N1 amplitudes over the left than over the right sensor cluster (left: *M* = −1.76, *SD* = 1.93; right: *M* = −0.45, *SD* = 2.46). Importantly, a significant interaction between reference and congruency was identified (see Tables 4 and 5 and Fig. 2). Post hoc analyses on participants’ own evaluations revealed larger N1 amplitudes for incongruent negative evaluations compared with congruent evaluations (*p* = .006) but not compared with incongruent positive evaluations (*p* = .058). Congruent and incongruent positive evaluations did not differ significantly (*p* = .359). Within evaluations about the interaction partner, no post hoc comparison reached significance (*p*s > .089). All further possible interactions also remained insignificant (*F*s < 2.98, *p*s > .091; see Tables 4 and 5).

#### EPN (260–360 ms)

For the EPN, significant main effects of reference (see Tables 4 and 5 and Fig. 2) and congruency were found. Regarding the main effect of reference, larger EPN amplitudes were elicited for self-related compared with other-related evaluations (self: *M* = −2.21, *SD* = 2.44; other: *M* = −1.89, *SD* = 2.33). For congruency, larger EPN amplitudes (incongruent negative: *M* = −2.09, *SD* = 2.29; *M* = −1.82, *SD* = 2.48; incongruent positive: *M* = −2.25, *SD* = 2.51) were found for incongruent positive compared with congruent evaluations (*p* = .004) but not compared with incongruent negative evaluations (*p* = .077). Congruent and incongruent negative evaluations did not differ significantly (*p* = .230). There was no main effect of channel-group laterality, *F*(1, 45) < 0.01, *p* = .960, η_*p*_² < .001.

Importantly, we identified an interaction between reference and congruency again (see Tables 4 and 5 and Fig. 2). Post hoc analyses within self-related evaluations showed larger EPN amplitudes for incongruent positive (*p* < .001) and incongruent negative (*p* = .002) compared with congruent evaluations. These incongruent positive and negative judgments did not differ significantly (*p* = .655). In other-related evaluations, no post hoc comparison reached significance (*p*s > .210). Additionally, there were significant interactions between congruency and laterality, *F*(2, 90) = 4.24, *p* = .017, η_*p*_² = .086, and between reference and laterality, *F*(1, 45) = 5.60, *p* = .022, η_*p*_² = .111. Effects of reference and congruency were not significant over left sensors—reference: *F*(1, 45) = 2.37, *p* = .131, η_*p*_² = .050; congruency: *F*(2, 90) = 0.99, *p* = .377, η_*p*_² = .021—but reached significance over right sensors—reference: *F*(1, 45) = 17.86, *p* < .001, η_*p*_² = .284; congruency: *F*(2, 90) = 9.21, *p* < .001, η_*p*_² = .170 (see Fig. 2). Finally, the remaining three-way interaction among reference, congruency, and laterality remained insignificant, *F*(2, 90) = 1.63, *p* = .201, η_*p*_² = .035.

#### Post hoc analyses N1-EPN (130–400 ms)

We explored a single time interval starting with the N1 and extending into the EPN window. We found a main effect of reference, *F*(1, 45) = 7.75, *p* = .008, η_*p*_² = .147, with more negative amplitudes for self-referent evaluations. There was a main effect of congruency, *F*(2, 90) = 6.26, *p* = .003, η_*p*_² = .122, with larger negative amplitudes for incongruent negative (*p* = .02) and incongruent positive (*p* = .001) evaluations compared with congruent evaluations. Finally, we observed an interaction of reference and congruency, *F*(2, 90) = 5.42, *p* = .006, η_*p*_² = .107. Within self-referent evaluations, incongruent negative (*p* < .001) and incongruent positive (*p* < .001) evaluations elicited a larger occipital negativity compared with congruent evaluations. All other comparisons were nonsignificant (*p*s > .171).

#### FRN (200–280 ms)

**Fig. 3.**
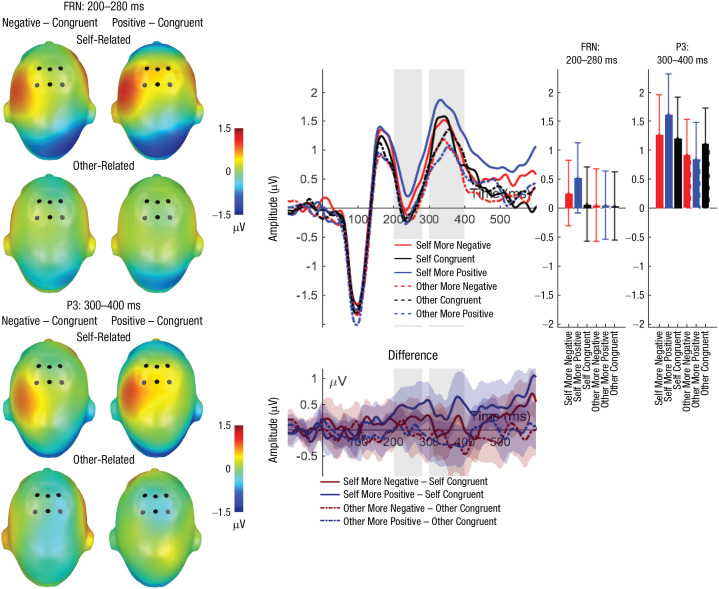
Feedback-related negativity (FRN): main effects of reference and frontal P3 interaction effects of reference and evaluation congruency. Scalp topographies (left) depict differences between the negative and congruent conditions and positive and congruent conditions, separately for self-related and other-related evaluations. Black dots indicate positions of electrodes. The waveform graph (top middle) shows the time course of event-related potentials (ERPs) over the depicted average FRN/P3 cluster, separately for incongruent positive, incongruent negative, and congruent self- and other evaluations. The gray shaded areas highlight the range of the FRN and P3, respectively. The bar charts (right) show mean amplitudes for each evaluation type, separately for the FRN and P3. Error bars represent 95% confidence intervals. The difference waveforms (bottom middle) show 95% bootstrapped confidence intervals of intra-individual differences in comparisons of interest.

For the FRN, a significant main effect of reference (see Tables 4 and 5 and Fig. 3) was observed; more positive FRN amplitudes were elicited for self-related compared with other-related evaluations (self: *M* = 0.31, *SD* = 1.99; other: *M* = 0.08, *SD* = 1.95). There was no significant main effect of congruency and no significant interaction effect of reference and congruency (see Tables 4 and 5). Exploratory analyses within the reference conditions showed that self-related incongruent positive evaluations elicited a larger positivity compared with both congruent evaluations (*p* = .011) and incongruent negative evaluations (*p* = .042), which did not differ significantly (*p* = .903). Within other-related evaluations, no differences occurred (*p*s > .873).

#### Frontal feedback P3 (300–400 ms)

Regarding the frontal feedback P3, a significant main effect of reference was found (see Tables 4 and 5 and Fig. 3); P3 amplitudes were greater for self-related compared with other-related evaluations (self: *M* = 1.38, *SD* = 2.28; other: *M* = 0.99, *SD* = 2.01). In contrast, no significant main effect of congruency was identified. Importantly, the interaction between reference and congruency reached significance (see Tables 4 and 5 and Fig. 3). Post hoc analyses within self-related evaluations showed larger P3 amplitudes for incongruent positive evaluations compared with both congruent evaluations (*p* = .035) and incongruent negative evaluations (*p* = .040); the latter two conditions did not differ significantly (*p* = .767). No differences were found within other-related evaluations (*p*s > .127).

#### LPP (500–750 ms)

**Fig. 4.**
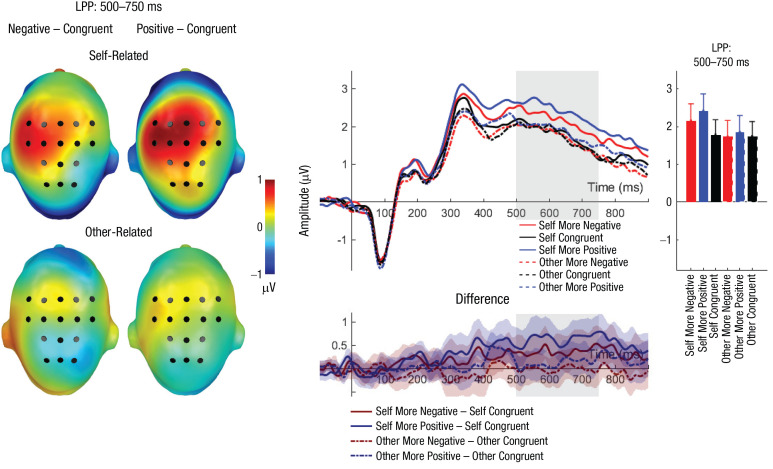
Late positive potential (LPP): main effects and interaction effects of reference and evaluation congruency. Scalp topographies (left) depict differences between the negative and congruent conditions and positive and congruent conditions, separately for self-related and other-related evaluations. Black dots indicate positions of electrodes. The waveform graph (top middle) shows the time course of event-related potentials (ERPs) over the depicted average LPP cluster, separately for incongruent positive, incongruent negative, and congruent self- and other evaluations. The gray shaded area highlights the range of the LPP. The bar chart (right) shows mean amplitudes for each evaluation type. Error bars represent 95% confidence intervals. The difference waveforms (bottom middle) show 95% bootstrapped confidence intervals of intra-individual differences in comparisons of interest.

For the LPP, significant main effects of reference and congruency were detected (see Tables 4 and 5 and Fig. 4). Here, larger LPP amplitudes were found for self-related compared with other-related evaluations (self: *M* = 2.11, *SD* = 1.32; other: *M* = 1.78, *SD* = 1.29) and for incongruent positive evaluations compared with congruent evaluations (*p* = .003) but not compared with incongruent negative evaluations (*p* = .085; incongruent negative: *M* = 1.94, *SD* = 1.40; congruent: *M* = 1.78, *SD* = 1.21; incongruent positive: *M* = 2.12, *SD* = 1.40). Congruent and incongruent negative evaluations did not differ significantly (*p* = .165). Importantly, there was a significant interaction between reference and congruency (see Fig. 4). Post hoc analyses on self-related evaluations revealed larger LPP amplitudes for both incongruent positive (*p* < .001) and incongruent negative (*p* = .034) evaluations compared with congruent evaluations, but they did not differ significantly from each other (*p* = .094). For other-related evaluations, no significant differences were found (*p*s > .436).

#### Control analyses

In control analyses, we found a main effect of congruency; there were more trials for incongruent negative and positive trials than congruent trials. Thus, we tested whether this imbalance of trial numbers influenced ERP differences. First, we calculated analyses with matched trial numbers per congruence condition, resulting in highly similar ERP effects; the LPP interaction was not significant (see Section A in the Supplemental Material). Second, to test whether an imbalance of total trial numbers during the experiment, rather than an imbalance in available ERP trials, affected ERP effects (see the first section in Results), we correlated the difference between incongruent and congruent total trials with ERP differences. We found no relationship between differences in the total number of available trial and ERP differences for the EPN (*r* = .140, *p* = .352, *N* = 46) but a negative relationship with the LPP (*r* = −.302, *p* = .041, *N* = 46).

## Discussion

This study examined ERPs in response to social evaluations based on real social interactions. Specifically, we investigated the effects of self-relevance and of congruency between participants’ own evaluations and their partner’s judgments. Our results showed that the self-related evaluations increased ERP amplitudes from early time points (N1) throughout midlatency (FRN, EPN) and late time windows (feedback P3, LPP). Furthermore, incongruence led to larger EPN, feedback P3, and LPP amplitudes. Most importantly, interactions between self-relevance and congruence occurred throughout the entire processing cascade. At early, sensory-related processing stages (N1), self-related incongruent negative evaluations differed significantly from congruent ones. Subsequently, both incongruent negative and positive self-related evaluations increased EPN and LPP responses. Finally, a selective amplification for self-related incongruent positive evaluations was detected for the feedback P3. In the following, we will discuss this selective modulation of all ERP components in order across the processing cascade.

From the N1 onward, amplitudes for self-related evaluations increased, which is in line with the *self-reference effect* (Symons & Johnson, 1997). Additionally, larger amplitudes were already observed at the N1 for participants’ incongruent negative self-evaluations. Such an enhancement of the N1 possibly reflects an early visual amplification of socially relevant information. This in line with the results of several other studies that have found enlarged amplitudes for socially significant decisions made by an interaction partner (Baess & Prinz, 2015; Schindler, Miller, & Kissler, 2019). Moreover, the N1 has recently been suggested as a marker of threat sensitivity in a two-generation study on social anxiety disorder (Harrewijn et al., 2018). In this regard, the enlarged N1 amplitudes found for self-related incongruent negative feedback in our study could be explained by the enhanced processing of self-threatening feedback. These interactions suggest that mismatches between participants’ own evaluations and the evaluations made by their interaction partners were detected at an early time point and seem to reflect a sustained increased negativity, showing a similar pattern in the EPN time window. This possibly reflects initial and sustained effects of updating and comparison processes for the integration of self-views and other views (Shrauger & Schoeneman, 1979).

Related to N1 interaction effects, during the EPN, both incongruent negative and positive evaluations were amplified when evaluations were related to participants’ own evaluations. These findings are consistent not only with the results of a number of studies showing self-relevance effects (e.g., Bayer et al., 2017) but also with the findings of social-feedback studies revealing stronger responses to emotionally relevant evaluations (e.g., Schindler et al., 2015). The increased early negativities are in line with early attentional-selection processes (see Schupp et al., 2004).

In the same time window, a main effect of self-reference was found for the FRN, which showed higher positivity for self-related evaluations. Although we found no main effect of congruency and no interaction, exploratory analyses showed more positive FRN amplitudes for self-related incongruent positive compared with congruent or incongruent negative feedback. Previous studies found larger FRN negativities for social rejections (e.g., Kujawa et al., 2014, 2017), and some mostly found an effect of feedback expectedness (van der Molen et al., 2017; Veen et al., 2016). One explanation might be that self-related positive evaluations are highly expected compared with all other conditions, in line with strong self-positivity biases (e.g., Korn et al., 2012; Sharot & Garrett, 2016). A second explanation might be that as this is the first study realizing a sort of baseline comparison (i.e., a congruent condition), this enables us to frame the findings into the large body of research relating the FRN as a reward positivity (Becker et al., 2014; Proudfit, 2015).

We found an interaction of self-reference and congruency here for subsequently peaking feedback P3 (see Fig. 3): Self-related positive information led to a pronounced frontal positivity that differed from all other conditions. This is consistent with recent findings showing large P3 amplitudes for positive evaluations, which previous research has suggested may represent a reward-related positivity (e.g., Pfabigan et al., 2011). Remarkably, we also observed that participants’ congruent and incongruent negative self-evaluations induced a larger feedback P3 than any evaluation about the interaction partner, which necessitates an extension of the above-mentioned hypothesis. Either self-related evaluations induce a higher global magnitude of feedback signaling (Osinsky et al., 2012; Pfabigan et al., 2011), or evaluations about oneself are rewarding per se. In line with the latter assumption, results of other research have shown larger striatal activations for social feedback per se (Schindler, Kruse, et al., 2019) and even for putative interactions with another human, regardless of the outcome (Pfeiffer et al., 2014).

Finally, we also found that self-related incongruent negative and positive evaluations led to larger LPP amplitudes (but see Section A in the Supplemental Material). As expected, self-related incongruent positive evaluations elicited the largest late positivities. These results are consistent with the findings of previous studies showing increased LPPs for self-relevant contexts (e.g., Herbert et al., 2011) and proposing a high relevance of social evaluations (e.g., Schindler, Miller, & Kissler, 2019). This is in line with the processes occurring during the late LPP time window, including stimulus evaluation, affective labeling, and controlling of self-related and emotion-regulation processes (see Hajcak et al., 2010; Schupp et al., 2006). In this regard, it is likely that the LPP reflects the suggested positively biased self-updating, leading to a more elaborative processing of self-serving information (e.g., Korn et al., 2012; Sharot & Garrett, 2016). In neuroimaging studies, self-view and self-updating processes have been associated with the activation of midline posterior areas (e.g., see Lieberman, 2007). Indeed, recent functional MRI findings show pronounced activations for self-relevant social evaluations in these regions (posterior cingulate cortex and precuneus; Schindler, Kruse, et al., 2019). Interestingly, these brain areas are also activated when participants have to give self-serving favorable self-evaluations after receiving social-rejection feedback (Hughes & Beer, 2012). This medial posterior activation has been associated with a protective strategy to maintain a positive self-view (Hughes & Beer, 2012). Of note, control analyses revealed that with increasing numbers of incongruent evaluations, LPP differences decreased, underestimating the LPP effects.

### Constraints on generality and outlook

In this study, we employed a novel design reporting neural responses during social interactions in a large sample. Our results clearly show that self-related and incongruent evaluations modulate neural processes at early and late stages. One limitation of this study is the naturalistic context with reduced experimental control (described above). However, in this dyadic approach we were able to develop a paradigm in which real social evaluations resulted in an equal proportion of self- compared with other-related and incongruent positive compared with negative evaluations. Further, with the limited number of positive and negative words (60 each), we could not perform any fine-grained analyses of how word valence affected incongruence.

Our results here might even underestimate the impact of self-related incongruent evaluations for two reasons. First, self-verification is proposed to be a central human motive, and therefore receiving congruent ratings about oneself should be rewarding per se (Kwang & Swann, 2010). Second, as people typically exhibit a strong social interest in others and compare themselves with others (Swencionis & Fiske, 2014), even other-related evaluations might have elicited intense electrophysiological responses. In our view, a promising direction for future studies is to collect, next to self-evaluations, participants’ predictions of how they expect to be evaluated by another participant, because self-evaluations do not necessarily match people’s expectations of how they are seen by others. Further, such predictions might change during the experiment. If indeed there are mismatches between expectations and self-evaluations, these can inform how one or the other drive specific modulations (e.g., FRN) in socioevaluative settings.

### Conclusion

For the first time, we investigated ERPs toward social evaluations after a real social interaction. We showed that evaluations of oneself increase early and late components of the ERP. Self-related incongruence is detected already at early processing stages (N1). Although a bias toward incongruent negative evaluations was initially found, at midlatency stages (EPN), both incongruent negative and positive self-evaluations were amplified, eventually leading to a more pronounced processing of incongruent positive self-evaluations (feedback-related P3, LPP). Thus, in real social interactions, evaluative feedback about oneself, and especially incongruent positive evaluations that violate one’s self-view, appear to modulate all processing stages, suggesting a strong social motive.

## Supplemental Material

sj-docx-1-pss-10.1177_0956797621995197 – Supplemental material for Let’s Talk About Each Other: Neural Responses to Dissenting Personality Evaluations Based on Real Dyadic Interactions Click here for additional data file. Supplemental material, sj-docx-1-pss-10.1177_0956797621995197 for Let’s Talk About Each Other: Neural Responses to Dissenting Personality Evaluations Based on Real Dyadic Interactions by Sebastian Schindler, Anne Höhner, Robert Moeck, Maximilian Bruchmann and Thomas Straube in Psychological Science

## Transparency

*Action Editor:* M. Natasha Rajah

*Editor:* Patricia J. Bauer

*Author Contributions*

A. Höhner, S. Schindler, and T. Straube designed the study. R. Moeck programmed the experiment. A. Höhner and S. Schindler pilot-tested the design and tested participants. A. Höhner, M. Bruchmann, and S. Schindler analyzed the data. S. Schindler wrote the manuscript under the supervision of T. Straube. All authors read and approved the final manuscript for submission.

**Open Practices:** All data have been made publicly available via OSF and can be accessed at https://osf.io/cfts6/. The hypotheses, experimental design, and analysis plan were preregistered at https://osf.io/9eqvy. This article has received the badge for Preregistration. More information about the Open Practices badges can be found at http://www.psychologicalscience.org/publications/badges.
