# Supplementary material for: Let’s Talk About Each Other: Neural Responses to Dissenting Personality Evaluations Based on Real Dyadic Interactions
Source: Psychol Sci. 2021 Jun 8;32(7):1058–72. doi: 10.1177/0956797621995197 (PMC8641130; doi:10.1177/0956797621995197)
Supplement: sj-docx-1-pss-10.1177_0956797621995197 – Supplemental material for Let’s Talk About Each Other: Neural Responses to Dissenting Personality Evaluations Based on Real Dyadic Interactions [file sj-docx-1-pss-10.1177_0956797621995197.docx]

**Let's talk about each other: Neural responses to dissenting personality evaluations based on real dyadic interactions**

**-**

**SUPPLEMENTARY MATERIALS**

**Section A: ERP analyses with matched trial numbers**

Additionally, to test if unequal numbers of congruent vs incongruent evaluations were causing the ERP modulations, we matched trial numbers between congruent and incongruent trials. Here, for self- and other-related evaluations, we had each 26 incongruent negative, 26 congruent and 26 incongruent positive trials. Accordingly, there were no main effects of reference (*F*_(1,45)_ < 0.01, *p* = .968, η_P_² < .001), congruence (*F*_(2,90)_ = 0.03 *p* = .964, η_P_² = .001) and no interaction (*F*_(2,90)_ = 0.07, *p* = .934, η_P_² = .002), regarding included number of trials.

ERP analyses show similar results compared to the analyses with unequal trials (see Supplementary Table 1). Main effects of reference were found from the EPN onwards sustaining into the LPP, while main effects of congruence were observed for the EPN and LPP. For the N1, EPN and feedback-P3 significant interactions of reference and congruence were found. For main effects of reference, larger negative amplitudes for self-related evaluations were found for the EPN, larger positive amplitudes for the FRN, feedback-P3 and LPP. For the main effects of congruence, larger EPN and LPP amplitudes were observed for incongruent positive evaluations. Regarding interactions, both for the N1 and EPN, self-related incongruent negative and positive evaluations led to larger negativities. For the feedback-P3, self-related incongruent positive evaluations led to a larger positivity than congruent or incongruent negative evaluations.

**Supplementary Table S1. ANOVA results for all ERPs with matched trials**

| **ANOVAs** | *main effect reference* | | | *main effect congruence* | | | *interaction effect* | | |
| --- | --- | --- | --- | --- | --- | --- | --- | --- | --- |
|  | *F_(1, 45)_* | *p* | *η_P_²* | *F_(1, 45)_* | *p* | *η_P_²* | *F_(1, 45)_* | *p* | *η_P_²* |
| **P1** | 0.06 | .806 | .001 | 0.40 | .675 | .009 | 0.64 | .532 | .014 |
| **N1** | 2.38 | .130 | .050 | 1.96 | .148 | .042 | **4.26** | **.017** | **.087** |
| **EPN** | **13.35** | **.001** | **.229** | **4.80** | **.010** | **.096** | **4.30** | **.017** | **.087** |
| **FRN** | **4.62** | **.037** | **.093** | 1.90 | .156 | .040 | 1.35 | .266 | .029 |
| **feedback-P3** | **8.22** | **.006** | **.154** | 1.22 | .301 | .026 | **3.45** | **.036** | **.071** |
| **LPP** | **8.05** | **.007** | **.152** | **3.52** | **.034** | **.073** | 2.49 | .089 | .052 |
| *Post-hoc tests* | | | | | | | | | |
| **P1** | No significant main or interaction effects | | | | | | | | |
| **N1** | Interaction: self-related: Incongruent negative (*p* = .004) and positive (*p* = .042) > congruent | | | | | | | | |
| **EPN** | Main effects: self- > other-related (*p* = .001); incongruent positive > congruent (*p* = .004); Interaction: self-related incongruent negative (*p* = .007) and positive (*p* < .001) > congruent | | | | | | | | |
| **FRN** | Main effect: self- < other-related (*p* = .037) | | | | | | | | |
| **feedback-P3** | Main effects: self- > other-related (*p* = .006); Interaction: self-related incongruent positive > congruent (*p* = .015) and incongruent negative (*p* = .032) | | | | | | | | |
| **LPP** | Main effects: self- > other-related (*p* = .007); incongruent positive > congruent (*p* = .016) | | | | | | | | |

Note. For post-hoc comparisons, only significant differences (*p* < .05) are reported. Direction is according to the functional meaning of the given ERP component.

**Section B: ANOVA comparing own and other evaluations (i.e. feedback)**

Additionally, we preregistered to report analyses on all trials, including the self-generated rating (see Supplementary Figure S1, highlighted in blue) and the evaluations from the other participant (see Supplementary Figure S1, highlighted in red). Own and other evaluations are mirrored across dyads, thus these analyses are based on the very same stimuli, except the reference who did the evaluation. However, they suffer from a temporal confound, as own ratings are always presented first and the evaluation from the other participant, which contains new information, is presented afterwards (see Supplementary Figure S1). In line with our predictions for the main analyses (see the main text), we firstly, predicted that self-related evaluations should lead to larger early and late ERPs. We also expected that evaluations from the other participant compared to evaluations given by oneself should increase amplitudes across the whole time window, as these are analyses below show that effects are based strongly on the feedback sent by the other participant.

**Preprocessing.** For all evaluations, self-generated own and those given by the interaction partner, on average, 84 percent trials were kept with no differences in kept trials between evaluation feedback source (own evaluation vs other evaluation; *F*_(1, 45)_ = 1.90, *p* = .175, η_P_² = .040), reference (self-referent vs other-referent; *F*_(1, 45)_ = 0.79, *p*= .380, η_P_² = .017), and emotion (negative vs positive adjective; *F*_(1, 45)_ = 0.02, *p*= .884, η_P_² < .001), as well as no interaction between these factors were found (*Fs <* 1.54, *ps* > .221). Here, on average for own generated self-referent evaluations, 49.76 trials for negative adjectives and 50.07 trials for positive adjectives were kept. For own generated other-referent evaluations, 50.54 trials for negative adjectives and 50.50 trials for positive adjectives were kept. For evaluations from the other person about oneself, 50.96 trials for negative adjectives and 50.54 trials for positive adjectives were kept. For evaluations from the other person about themselves, 50.85 trials for negative adjectives and 50.76 trials for positive adjectives were kept.

**
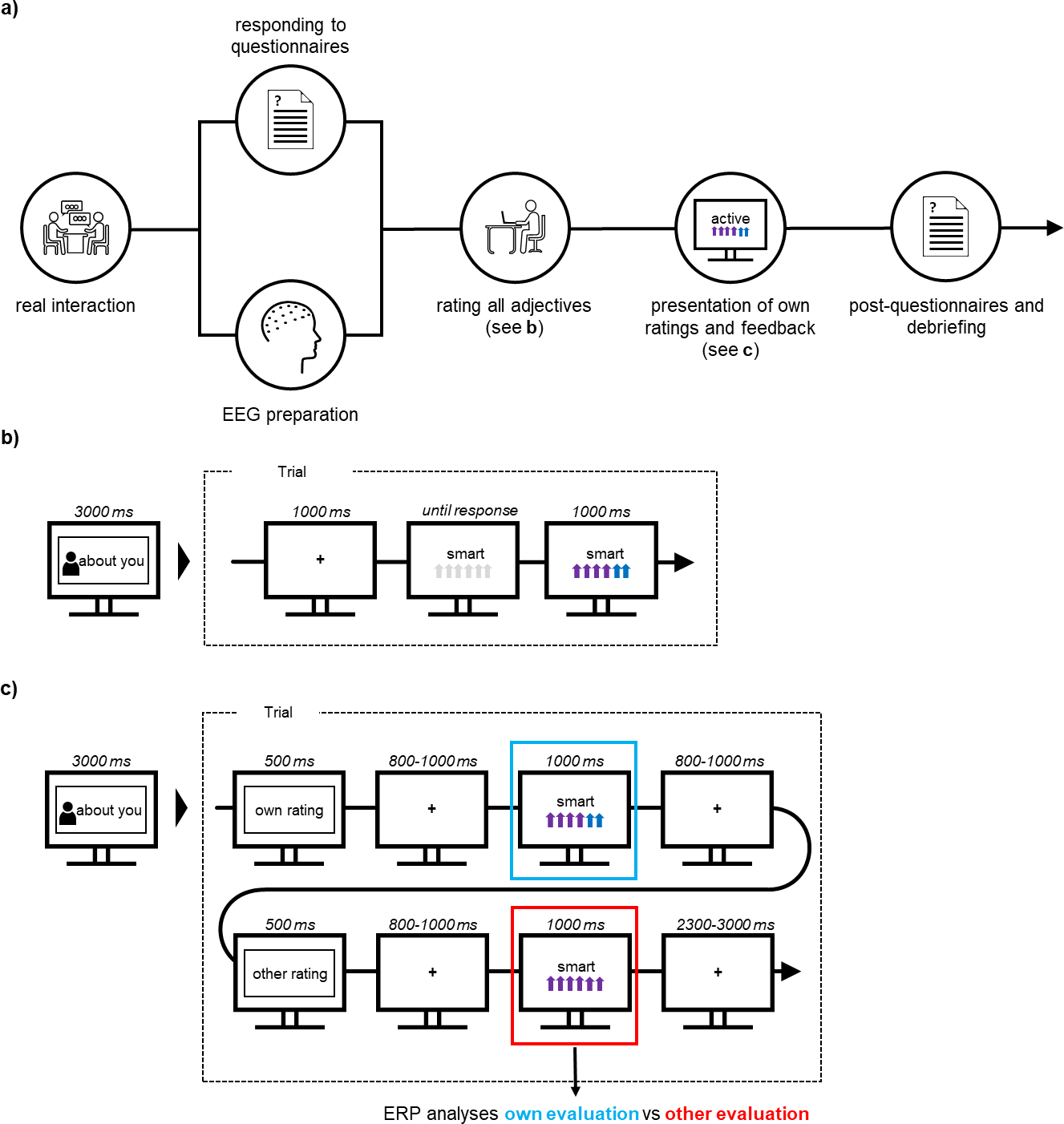
 Supplementary Figure S1: Experimental design (a) and trial design (b and c). (a)** The complete time schedule of the experiment. **(b)** The trial design of collecting self- and interaction partner ratings while EEG was prepared. **(c)** Structure of the main experiment, with the own ratings presented first, followed by the rating decisions made by the interaction partner. For completeness, we also analyzed the representation of own evaluations (blue) compared to evaluations given by the interaction partner (red) having perceptual identical but mirrored trials across dyads. However, own ratings are always presented first.

**Statistical analyses.** The full model compared all evaluations, having two (evaluation sender: own evaluations, other (feedback) evaluations), by two (reference: self-referent, other-referent), by two (word emotion: negative, positive adjectives) Repeated Measures ANOVAs, investigating main effects of sender, reference, emotion, and their interactions. Here, time windows and electrode clusters matched the analyses in the main text.

**ERP results for all evaluations: own-evaluation vs other-evaluations**

**P1 (80-100ms)**

For the P1 a significant main effect of evaluation sender was observed (see Supplementary Table S2, Supplementary Figure S2), but no main effects of reference or emotion. Larger P1 amplitudes were found for evaluations by the other participant. Further, a three-way interaction between evaluation sender, reference, and emotion occurred. However, resolving this interaction, both the two-way interactions between reference and emotion for own evaluations was insignificant (*F*_(1, 45)_ = 2.32, *p* = .135, η_P_² = .049) and the analogous two-way interaction for evaluations by the other participant was insignificant as well (*F*_(1, 45)_ = 1.57, *p* = .217, η_P_² = .034). All other interactions were insignificant (see Supplementary Table S2).

**Supplementary Table S2. ANOVA results for all evaluations and all examined ERP components**

| **ANOVAs** | *main effect evaluation sender* | | | *main effect reference* | | | *main effect emotion* | | |  | | | |
| --- | --- | --- | --- | --- | --- | --- | --- | --- | --- | --- | --- | --- | --- |
|  | *F_(1, 45)_* | *p* | *η_P_²* | *F_(1, 45)_* | *p* | *η_P_²* | *F_(1, 45)_* | *p* | *η_P_²* |  |  |  |  |
| **P1** | **9.31** | **.004** | **.171** | 1.81 | .185 | .039 | 1.26 | .268 | .027 |  |  |  |  |
| **N1** | 0.48 | .490 | .011 | 2.56 | .117 | .054 | 0.61 | .441 | .013 |  |  |  |  |
| **EPN** | **81.43** | **<.001** | **.644** | **8.48** | **.006** | **.159** | 0.40 | .529 | .009 |  |  |  |  |
| **FRN** | 0.77 | .385 | .017 | **4.11** | **.048** | **.084** | 0.45 | .507 | .010 |  |  |  |  |
| **feedback-P3** | **37.47** | **<.001** | **.454** | **5.37** | **.025** | **.107** | 3.83 | .056 | .078 |  |  |  |  |
| **LPP** | **48.47** | **<.001** | **.519** | **7.62** | **.008** | **.145** | **4.54** | **.039** | **.092** |  |  |  |  |
| **ANOVAs** | *two-way interaction sender*reference* | | | *two-way interaction sender*emotion* | | | *two-way interaction reference*emotion* | | | *three-way interaction sender*reference*emo* | | | |
|  | *F_(1, 45)_* | *p* | *η_P_²* | *F_(1, 45)_* | *p* | *η_P_²* | *F_(1, 45)_* | *p* | *η_P_²* | *F_(1, 45)_* | *p* | *η_P_²* |  |
| **P1** | 0.53 | .471 | .012 | <0.01 | .986 | .001 | <0.01 | .996 | .001 | **4.37** | **.042** | **.088** |  |
| **N1** | 1.59 | .214 | .034 | 2.46 | .124 | .052 | <0.01 | .951 | .001 | 1.53 | .222 | .033 |  |
| **EPN** | **7.50** | **.009** | **.143** | <0.01 | .989 | <.01 | 0.02 | .904 | .001 | 0.21 | .648 | .005 |  |
| **FRN** | 3.91 | .054 | .080 | 1.62 | .210 | .035 | 0.18 | .676 | .004 | 2.13 | .152 | .045 |  |
| **feedback-P3** | **9.23** | **.004** | **.170** | **5.12** | **.028** | **.102** | 0.88 | .353 | .019 | 1.75 | .193 | .037 |  |
| **LPP** | 3.63 | .063 | .075 | 0.17 | .684 | .004 | 0.22 | .642 | .005 | 0.97 | .331 | .021 |  |

Please Note. Significant main and interaction effects are highlighted by bold font.

**N1 (130-180ms)**

For the N1, no main effects and no interaction effects were observed (see Supplementary Table S2, Supplementary Figure S2).

**EPN (260-360ms)**

For the EPN, a large main effect of evaluation sender was found (see Supplementary Table S2, Supplementary Figure S2), showing larger EPN amplitudes for evaluations by the other participant. Further, a main effect of reference showed larger negative waves were found for self-referent evaluations. No main effect of emotion was detected. There was also a two-way interaction between sender and reference, showing that the difference between self-related compared to other-related evaluations were larger when the evaluations were given by the other participant (*p* = .003; see Supplementary Figure S2). All other interactions were insignificant (see Supplementary Table S2).

**FRN (200-280ms)**

For the FRN a main effect of reference was found, showing a larger negativity for evaluations about the other participant. All other main effects and interaction effects were not significant (see Supplementary Table S2, Supplementary Figure S2).

**
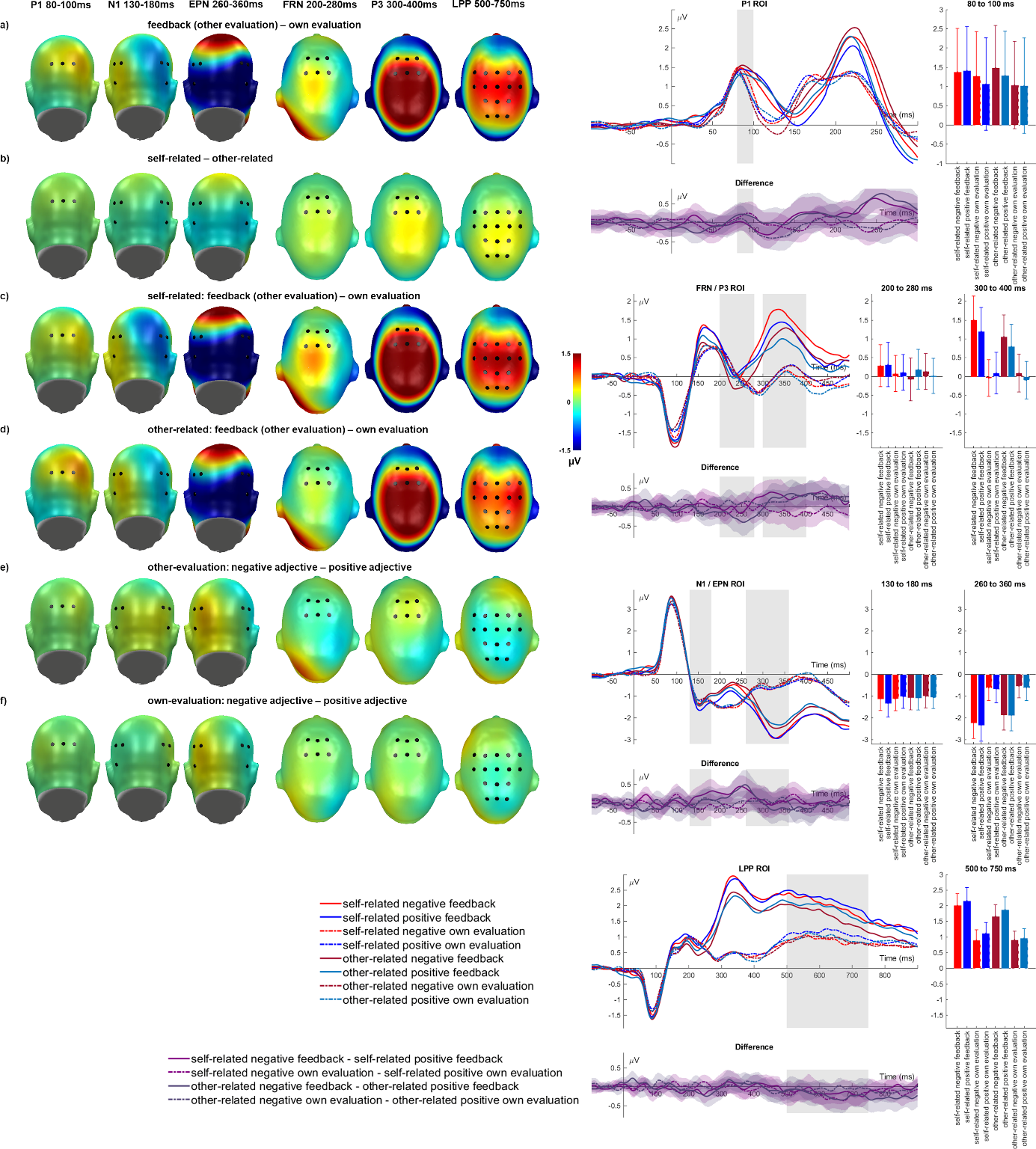
 Supplementary Figure S2: For all ERP components, scalp topographies depict differences for the main effects of evaluation sender (a), reference (b) and interactions of sender by reference (c-d) and sender by emotion (e-f).** The ERPs (P1, N1, EPN, FRN, P3, LPP) show the time course over the depicted average electrode cluster. For bar charts, error bars show 95 % confidence intervals. The difference plots contain 95 % bootstrap confidence intervals of intra-individual differences.

**Frontal feedback-P3 (300-400ms)**

For the frontal P3, we found a large main effect of evaluation sender was found (see Supplementary Table S2, Supplementary Figure S2), showing larger P3 amplitudes for evaluations by the other participant. Further, a main effect of reference showed larger P3 amplitudes for self-referent evaluations. No main effect of emotion was detected. There was a significant two-way interaction between sender and reference, showing that the difference between self-related compared to other-related evaluations were larger when the evaluations were given by the other participant (*p* = .004; see Supplementary Figure S2). Additionally, a significant two-way interaction between sender and emotion was found. Here, a difference between negative and positive adjectives was larger when evaluations were given by the other participant (*p* = .028). All other interactions were insignificant (see Supplementary Table S2).

**LPP (500-750ms)**

For the LPP, separate significant main effects of evaluation sender, reference, and emotion were found, showing larger positive amplitudes for evaluations by the other participant compared to self-generated evaluations, self-related compared to other-related evaluations and for positive compared to negative adjectives (see Supplementary Figure S2). All interactions were insignificant (see Supplementary Table S2).
